# Blastocyst transfer does not improve cycle outcome as compared to D3 transfer in antagonist cycles with an elevated progesterone level on the day of hCG

**DOI:** 10.4274/jtgga.2017.0012

**Published:** 2017-09-01

**Authors:** Cem Demirel, Serkan Aydoğdu, Arzu İlknur Özdemir, Gülşah Keskin, Ercan Baştu, Faruk Buyru

**Affiliations:** 1 In Vitro Fertilization Center, Memorial Ataşehir Hospital, İstanbul, Turkey; 2 Department of Obstetrics and Gynecology, İstanbul University School of Medicine, İstanbul, Turkey

**Keywords:** Blastocyst transfer, human chorionic gonadotropin administration, progesterone elevation

## Abstract

**Objective:**

To evaluate the association between progesterone elevation on the day of human chorionic gonadotropin (hCG) administration and clinical pregnancy rates of gonadotropin-releasing hormone (GnRH) antagonist in vitro fertilization/intracytoplasmic sperm injection (IVF/ICSI) cycles with the transfer of embryos at different developmental stages (day-3 versus day-5 ETs).

**Material and Methods:**

This is a retrospective analysis of fresh IVF/ICSI; 194 cycles out of 2676 conducted in a single center.

**Results:**

A total of 2676 cycles were analyzed, of which 386 had no progesterone measurements available. Two hundred eighteen cycles had progesterone elevation (p>1.5 ng/mL) giving an overall incidence of 9.5%. Twenty-four cycles were excluded from further analysis. Of the remaining 194 cycles, 151 had day-3 transfers and 43 had blastocyst transfers. There was no statistically significant difference in pregnancy and clinical pregnancy rates per transfer between the D3-ET and D5-ET groups (46% vs. 49%, and 39% vs. 35%, respectively).

**Conclusion:**

The results of this study suggest that blastocyst transfer does not improve cycle outcomes compared with D3 transfer in GnRH antagonist cycles with an elevated progesterone level on the day of hCG.

## INTRODUCTION

Attention has been extensively paid during the last 20 years to serum progesterone measurement during ovarian stimulation. Since the early 90s, many studies have documented that a premature and excessive progesterone elevation above a certain threshold, before triggering of ovulation, might negatively affect of in vitro fertilization (IVF) outcomes (1, 2, 3).

Elevated progesterone (P_4_) levels in the follicular phase has been a matter of debate in terms of IVF cycle outcomes. Apart from the discrepancies in definition of “elevated P_4_,” some studies claimed no effect at all (4, 5, 6), whereas others reported poorer outcomes (7, 8, 9, 10, 11, 12). A favorable effect on pregnancy rates was also documented in an earlier study (13).

It was reported for the first time in 1991 that serum progesterone may increase during the last few days of ovarian stimulation (14). This has been widely confirmed during the last two decades, but the incidence of progesterone elevation greatly varies between published studies (2-35%) (15, 16).

This increase does not reflect “premature luteinization”. Progesterone elevation occurs because the risk of endogenous leutinizing hormone (LH) surge is usually controlled by simultaneous administration of gonadotropin-releasing hormone (GnRH) analogues or antagonists. Progesterone elevation during ovarian stimulation is primarily related to the intensity of the ovarian response to follicle-stimulating hormone (FSH), but is also dependent on the studied population, which may consist of normal or good repsonders.

It has been supposed that premature elevation of P_4_ advances the endometrium and leads to embryo-endometrial asynchrony. High serum P_4_ levels on the day of human chorionic gonadotropin (hCG) administration induce both advanced endometrial histologic maturation and differential endometrial gene expression, which decrease endometrial receptivity and might be related to implantation failure (17, 18). Progesterone elevation prematurely opens the window of implantation, modifies endometrium receptivity, and is associated with a defective implantation. A management strategy with robust evidence is lacking in such cycles. Postponing embryo transfers (ET) may result in better synchronization between the embryo and the already ahead-of-phase endometrium.

Papanikolaou et al. (11) and Elgindy (9) concluded that elevated P_4_ had a detrimental effect on day-3 but not day-5 ETs. The authors suggested that on the fifth luteal day, the endometrium had sufficiently recovered to allow for normal implantation. On the other hand, Hill et al. (19), Huang et al. (20), Corti et al. (21), and Ochsenkühn et al. (10) suggested that progesterone elevation on the day of hCG triggering had a negative impact on IVF outcomes, even with blastocyst transfers. These results were contrary to the findings of previous studies (9, 11).

The aim of our study was to evaluate the association between progesterone elevation on the day of hCG administration and clinical pregnancy rates of GnRH antagonist IVF/ICSI cycles with the transfer of embryos at different developmental stages (day-3 vs. day-5 ETs).

## MATERIAL AND METHODS

### Subjects

This is a retrospective analysis of fresh IVF/intracytoplasmic sperm injections (IVF/ICSI); 194 cycles out of 2676 conducted from January 2006 to August 2011 in a single center. The study protocol was approved by the instutional review board of the hospital; informed consent was waived due to the retrospective nature of this study.

The study inclusion criteria were: (1) GnRH antagonist cycles with a P_4_>1.5 ng/mL on day of hCG, (2) in which ≥8 MII oocytes were retrieved and (3) at least three 8-cell embryos were present on day 3, (4) women were aged <40 years with regular cycles, (5) day-3 FSH level of <10 IU/L, antral follicle count of >5, and (6) an endometrial thickness ≥8 mm on the hCG day. Each patient was included in the study only once in the data set of the present study.

The exclusion criteria were: (1) use of frozen-thaw ETs, (2) previous history of poor ovarian response, (3) pre-implantation genetic diagnosis (PGD) cycles, (4) GnRH agonist- trigerred cycles, (5) the use of testicular sperm, (6) known endocrine disorders, (7) cases where blood was drawn and analyzed in another laboratory.

The following patient characteristics were assessed: cause of infertility, age, duration of gonadotropin stimulation, E2 and P_4_ levels on the hCG day, the numbers of oocytes retrieved, MII and 2PN fertilized oocytes, and transfered embryos.

### Controlled ovarian hyperstimulation protocol

The GnRH antagonist protocol was initiated on day 2 of the menstrual cycle with either hMG or rFSH (Menogon, Ferring, Switzerland or Gonal F 75 IU ampules; Serono, Geneva, Switzerland; 150-300 IU/d) for ovarian stimulation. The dose was adjusted for each patient according to the follicular growth detected using ultrasonography after the 5^th^ day of drug administration. GnRH antagonist Orgalutran (Organon, Netherlands) 0.25 mg/dL per day was started on stimulation day 5.

### Ovarian follicular development and oocyte retrieval

When at least two follicle were ≥18 mm, 10,000 IU hCG (Pregnyl SC freeze-dried ampoule, MSD, Baxter Pharmaceutical Solutions LLC, Bloomington, USA) or 250 µgr of rec-hCG (Ovitrelle, Serono, Germany) was administered to trigger ovulation.

Oocyte retrieval was performed at hour 35 after hCG injection. An oocyte pick-up was completed using a 17-gauge needle for oocyte retrieval under local anesthesia. The oocyte–corona complexes were denuded, intracytoplasmic sperm injection was performed after 2 hours of incubation, and embryos were transferred on days 3 or 5.

### Embryo transfer and luteal phase support

The fertilized oocytes were observed for morphology on day 3. One hundred fifty-one participants underwent ET on day 3 (D3-ET), and 43 underwent ET on day 5 (D5-ET) because there was an adequate number of high-quality embryos available. Only high-quality embryos were transferred both on D3-ET and D5-ET. The choice of the ET day was mainly based on the embryo morphology, clinicians’ preference for cryopreservation of spare embryos on day 3, and workload of the laboratory. Embryologists graded embryos as good, fair, or poor in line with the simplified Society for Assisted Reproductive Technology scoring system (22).

Both groups were tested for serum βhCG 12 days after ET and transvaginal ultrasound was scheduled 3 weeks afterwards to confirm clinical pregnancy. The luteal phase was supported with intravaginal micronized progesterone (Progestan 200 mg; Koçak, Tekirdağ, Turkey) as 600 mg/day, starting on the day of oocyte retrieval.

### Hormonal evaluation

On the day of hCG trigger, serum P_4_ and E2 levels were measured on a blood sample drawn at 10:00 AM. We used a microparticle enzyme immunoassay (Axsym System; Advia Centaur, Siemens), which has a sensitivity of 0.21 ng/mL. For the P_4_ assay, the intra- and interassay coefficients of variation are 7.2% and 5.7%, respectively. The E2 assay has a sensitivity of 7.0 pg/mL, with intra- interassay coefficients of variability of 11.3% and 5.0%, respectively. We selected a serum progesteron level of 1.5 ng/mL on the day of hCG administration as a cutoff level for an adverse cycle outcome as evidenced by the literature (9).

### Evaluation of in vitro fertilization/intracytoplasmic sperm injection results

Clinical pregnancy and early pregnancy loss rates of D3-ET and D5-ET groups were evaluated. Clinical pregnancy rate was the primary outcome. Clinical pregnancy was defined as the presence of a gestational sac on transvaginal sonography.

### Statistical analysis

The statistical analysis of the study was performed using the Statistical Package for the Social Sciences 20.0 (SPSS Inc., Chicago, IL, USA) and G*Power 3 (Düsseldorf, Germany) (23). Categorical variables in the data set are given with frequencies and percentages, but the continuously changing variables are given with mean, standard deviation, median, minimum and maximum values. The compliance of the measurement variables with normal distribution was analyzed using the Shapiro-Wilk test. In the comparison of two groups of variables with normal distribution, the difference between the two means was determined using the significance test (t test), and the comparison of variables that do not show normal distribution was performed using the Mann-Whitney U test. In the comparison of categorical variables between groups, Yates’s corrected Chi-square test was used. In all the statistical analyses in the study, comparisons under a p-value of 0.05 were considered statistically significant.

## RESULTS

A total of 2676 cycles were analyzed, of which 386 had no progesterone measurements available. Two hundred eighteen cycles were noted to have progesterone elevation (p>1.5 ng/mL) giving an overall incidence of 9.5%. Twenty-one cycles were excluded from further analysis because there were fewer than three 8-cell embryos on day 3 or no blastocysts on day 5. Three additional cycles were excluded because of total embryo freezing and no fresh ET in the given cycle. Of the remaining 194 cycles, 151 had day-3 transfers, and 43 had blastocyst transfers.

There was no statistically significant difference in the demographics between the two study groups (Table 1).

The mean age of D3-ET group was 30.65±4.12 years (range, 19-40 years), and that of the D5-ET group was 29.9±3.07 years (range, 22-36 years). Both groups had a similar duration of controlled ovarian hyperstimulation. The mean level of serum progesterone on the day of hCG administration was 1.83±0.49 ng/mL in the D3-ET group and 1.92±0.87 ng/mL in the D5-ET group (p>0.05). The D5-ET group had a significantly higher mean estradiol level on the day of hCG (3940.7±1928.20 vs. 2803.62±1639.73 pg/mL, p=0.001). The mean number of oocytes retrieved was significantly higher (22.23±8.93 vs. 15.63±7.76, p=0.001) in the D5-ET group, along with a higher number of MII (17.1±6.75 vs. 10.86±5.86, p=0.001) and 2PN fertilized oocytes (13.33±5.03 vs. 7.81±4.59, p=0.001), respectively. There was no statistically significant difference in the mean number of embryos transferred in the day 3 and day 5 groups (2.2 vs. 1.8, respectively) (Table 2).

**Figure 1:**
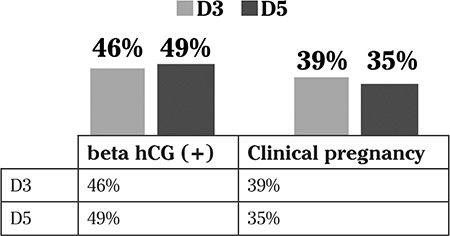
Pregnancy and clinical pregnancy rates per transfer between groups

There was no statistically significant difference in pregnancy and clinical pregnancy rates per transfer between the D3-ET and D5-ET groups (46% vs. 49%, and 39% vs. 35%, respectively) (Figure 1).

## DISCUSSION

In the present study, D5 transfer was not found to be superior to D3 transfer of embryos in patients with elevated P_4_ levels, respectively, when the cutoff level for P_4_ was set at 1.5 ng/mL on the day of hCG administration.

Elevated P_4_ in the late follicular phase of an IVF cycle is claimed to result in worse cycle outcomes. This negative effect is believed to be more prevalent in cycles with a higher oocyte yield; such a negative effect may ensue with a relatively higher P_4_ elevation. It is more likely that the elevated P_4_ levels reflect the total amount of progesterone secreted by maturing follicles, and these levels have been found to correlate positively with the number of mature follicles and with estradiol levels on hCG day. In the present study, we also documented an increase in E2 levels in correlation with number of mature follicles. Although non-significant, P_4_ levels were slightly elevated in the D5-ET group, in which the number of oocytes was significantly higher.

The first attempt to critically evaluate the existing literature regarding P_4_ elevation on the day of hCG and its role in pregnancy achievement was published in 2007 (6). The results of that review were confounded by the different GnRH analogue protocols administered. Moreover, the majority of the included studies that failed to demonstrate a negative association used an arbitrarily defined threshold value of 0.9 ng/mL. Following that meta-analysis, a prospective study by Elgindy (9) claimed that an increased P_4_ level of ≥1.5 ng/mL on hCG day was associated with an adverse effect on clinical outcomes. A meta-analysis of Kolibianakis et al. (24) evaluated the results of five eligible studies of GnRH antagonist cycles. They reported that women with elevated P_4_ level on the hCG administration day had decreased probability of clinical pregnancy per cycle. Another meta analysis of Venetis et al. (16) provided convincing data that elevation of serum P_4_ secretion was associated with low pregnancy rates whatever the GnRH analogue used. A very recent meta-analysis that reviewed only antagonist cycles documented that women with elevated P_4_ levels >1.5 ng/mL on hCG day had more oocytes and higher E2, as well as decreased probability of pregnancy per cycle (25).

The crucial question is how should physicians manage patients with elevated progesterone levels during late follicular phase. Proposed cycle management strategies in the event of high P_4_ on the day of hCG may be to freeze all embryos and transfer them back in a natural or hormone-replacement cycle, to favor a D5 ET, to start with a lower FSH dose in the next cycle, to use hp-hMG instead of rFSH, and/or earlier administration of hCG for triggering final oocyte maturation in high-risk patients. None of the aforementioned strategies have been tested so far for their efficacy in this setting.

For women with an elevated P_4_ level on the day of hCG, extending culture and transferring embryos on D5 might have been a sound strategy because the most probable mechanism of impairement that high P_4_ in the follicular phase causes is the advancement of endometrial maturation and early closure of the endometrial implantation window; day-5 ET may restore this asynchronization in such cycles.

In several studies it is claimed that endometrial advancement due to controlled ovarian hyperstimulation and raised P_4_ could be recovered on day 5 (26). Papanikolaou et al. (11) designed a study to determine if there was an effect of elevated P_4_ on hCG day on pregnancy outcomes, and whether this effect might be associated with the developmental stage of the embryo transferred. According to this study, even modest rises of P_4_ in the follicular phase has a detrimental effect on the implantation potential of good-quality cleavage stage embryos (11). On the contrary, premature luteinization in the blastocyst transfer subgroup had no effect on pregnancy outcomes.

Hill et al. (19) confirmed the recent publications by demonstrating a negative impact of elevated serum P levels on the day of hCG administration on live birth. This negative effect was also demonstrated in both cleavage and blastocyte stage ETs for both poor and good embryos. Huang et al. (20) reported that the negative association of P_4_ elevation with clinical pregnancy rates was noted both in D-3 and blastocyte stage ET cycles and confirmed decreased clinical pregnancy rates in GnRH agonist IVF/ICSI cycles regardless of the developmental stage of the transferred embryos. Ochsenkühn et al. (10) documented a similar reduction in pregnancy rates in the study of blastocyte transfers. However, their study had no cohort of cleaveage embryos to use for direct comparision. Corti et al. (21) and Ochsenkühn et al. (10) suggested that progesterone elevation on the day of hCG triggering has a negative impact on IVF outcomes, even with blastocyst transfers. These results were contrary to the findings of Papanikolaou et al. (11). The study of Papanikolaou addressed whether the adverse effect of follicular phase P_4_ elevation could be alleviated by a blastocyst transfer. In contrast to what this retrospective study suggested, in our data set, D5 transfer was not found as superior to D3 transfer of good quality embryos in patients with elevated P_4_ levels. Our study was retrospective in design but, the inclusion criteria enabled us to select patients with a cohort of good quality embryos on day 3 that might have a good chance of D5 transfer if they had been allowed to stay in extended culture. Thus, the two transfer groups presented a similar embryo development profile in culture. The reason for the lack of efficiency of D5 transfer strategy may be the fact that the advanced endometrium may still have not recovered from the action of elevated follicular P_4_. To our knowledge, there is no solid biologic evidence to confirm this endometrial recovery with concomittant D3 and D5 endometrial biopsies.

The choice of 1.5 ng/mL as a threshold level for P_4_ in our study is less than ideal because the threshold for poor cycle outcomes may change with the ovarian response of the patient. In better responders, a higher P_4_ threshold is more plausible. As such, the reason for failure of observing improved outcomes with a D5 ET strategy in our study may be that our patient population comprised either normal or good responder patients and thus a higher threshold could have revealed a positive treatment effect in favor of D5 transfer. The strongest effect of progesterone elevation on pregnancy rates was observed between 1.5 and 1.75 ng/mL in the study of Venetis et al. (16). Nevertheless, as the degree of ovarian response is increased, the failure of a beneficial effect for D5 ETs still may persist. In poor responders with an elevated P_4_ level, the management strategy could have been to freeze all embryos rather than to try a D5 transfer due to the lack of availability of an adequate number of embryos for extended culture.

The limitation of our study is its retrospective nature; this kind of study has selection bias. Also, the number of D5 transfers was lower than that of D3, which also weakned the study power.

In conclusion, the results of this retrospective study suggest that blastocyst transfer does not improve cycle outcomes as compared with D3 transfer in GnRH antagonist cycles with an elevated progesterone level on the day of hCG. Therefore, a prospective randomized control trial on an intention-to-treat basis is needed to compare single D3 and single blastocyst transfers in this setting to reach a more definitive answer to the problem.

## Figures and Tables

**Table 1:**
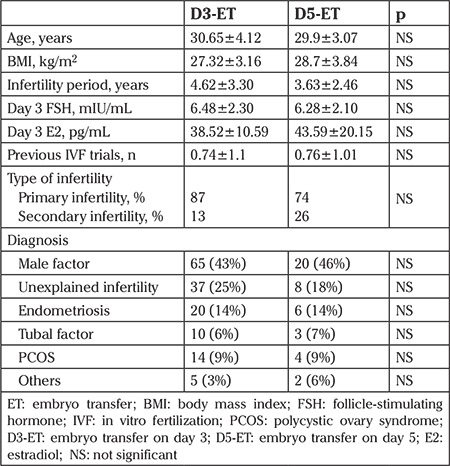
Patient overview

**Table 2:**
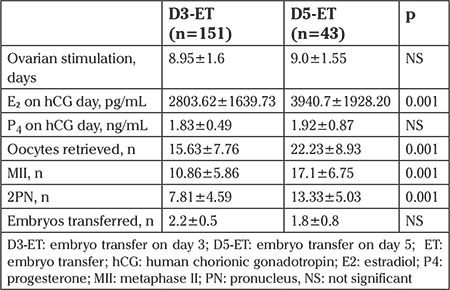
Cycle parameters in D3–ET and D5–ET groups in patients with P_4_ >1 ng/mL on the day of hCG
